# Potential clinical utility of MUC5B und TOLLIP single nucleotide polymorphisms (SNPs) in the management of patients with IPF

**DOI:** 10.1186/s13023-021-01750-3

**Published:** 2021-02-27

**Authors:** Francesco Bonella, Ilaria Campo, Michele Zorzetto, Eda Boerner, Shinichiro Ohshimo, Dirk Theegarten, Christian Taube, Ulrich Costabel

**Affiliations:** 1 Center for Interstitial and Rare Lung Diseases, Pneumology Department, Ruhrlandklinik University Hospital, University of Duisburg-Essen, 45239 Essen, Germany; 2 SC Pneumologia - Fondazione IRCCS Policlinico San Matteo, Pavia, Italy; 3 Clinical Chemistry Laboratory, Fondazione IRCCS Policlinico San Matteo, Pavia, Italy; 4 Department of Emergency and Critical Care Medicine, Graduate School of Biomedical and Health Sciences, Hiroshima University, Hiroshima, Japan; 5 Institute of Pathology, University Hospital Essen, University of Duisburg-Essen, Essen, Germany

**Keywords:** IPF, MUC5B, TOLLIP, Disease progression

## Abstract

**Background:**

Genetic variants of TOLLIP and MUC5B, both on chromosome 11, have been reported to be associated with the development and/or prognosis of idiopathic pulmonary fibrosis (IPF). This retrospective study was conducted to investigate the association of MUC5B and TOLLIP SNPs with disease outcome in IPF. 62 IPF patients and 50 healthy controls (HC) from our Institution were genotyped for SNPs within MUC5B (rs35705950) and TOLLIP (rs3750920 and rs5743890). Correlation of SNPs genotypes with survival, acute exacerbation (AE) or disease progression (defined as a decline of ≥ 5% in FVC and or ≥ 10% in DLco in one year) was investigated.

**Results:**

The MUC5B rs35705950 minor allele (T) was more frequent in IPF subjects than in HC (35% vs 9% *p* < 0.001). TOLLIP SNPs alleles and genotype distribution did not differ between IPF and HC and did not vary according to gender, age, BMI and lung functional impairment at baseline. The minor allele (C) in TOLLIP rs5743890 was associated with worse survival and with disease progression in all performed analyses. The MUC5B rs35705950 or the TOLLIP rs3750920 minor allele, were not associated with disease progression or AE.

**Conclusion:**

We confirm that the minor allele of MUC5B rs35705950 is associated with IPF. The minor allele of TOLLIP rs5743890 appears to be a predictor of worse survival and more rapid disease progression, therefore being of potential utility to stratify IPF patients at baseline.

## Background

Idiopathic pulmonary fibrosis is a chronic, progressing and ultimately fatal lung disease, characterized by loss of lung volume and development of respiratory insufficiency over time. Whereas lung functional and imaging parameters are currently used to monitor disease progression in the clinical routine, circulating biomarkers still have limited clinical impact due to issues with reproducibility and inter-individual variability [1]. On the other hand, data from genetic investigations in interstitial lung diseases, mainly in IPF, indicate that single nucleotide polymorphisms (SNPs) associated with the development of lung fibrosis correlate with disease outcome or response to certain treatments [2–4].

MUC5B affects the rheological properties of airway mucus, mucociliary transport, and airway defense [5]. The MUC5B promoter variant rs35705950 has been validated as a contributor to the expression of MUC5B in the lung [6]. This polymorphism may be related to the methylation in the MUC5B promoter region and the disruption of the activities of some transcriptional factors [7]. The minor allele (T) in rs35705950 SNP confers the risk of lung fibrosis [8] but patients with the TT genotype show better survival [3, 6]. It has been hypothesized that persistent bronchiolar epithelial injury and the over- production of MUC5B by airway progenitor cells result in the development of honeycomb cysts (UIP Pattern) and IPF [9].

Genetic variants of TOLLIP have also been reported to be associated with the development and/or prognosis of IPF [2]. The TOLLIP protein is an inhibitor of the toll-like receptors (TLRs) 2 and 4which both are active in the lung, thereby suppressing tumor necrosis factor-a (TNF-a) and IL-6 production [10]. The rs5743890 minor allele is associated with a 50% reduction in TOLLIP production whereas rs3750920, located within TOLLIP exon 3, has been linked to decreased TOLLIP mRNA expression and differential responses to treatment in IPF [4]. In particular, significant interaction was observed between N-acetylcysteine (NAC) therapy and rs3750920 within TOLLIP. A trend toward harm was observed in patients with a CC genotype treated with NAC, but not in those with a TT genotype, suggesting that a genotype-stratified prospective clinical trial should be conducted before any recommendation regarding the use of off-label NAC to treat IPF [4].

The aim of this study was to investigate the association of MUC5B and TOLLIP SNPs with disease course and outcome in IPF patients.

## Methods

### Study subjects

We retrospectively studied 62 patients with IPF followed in our Institution from 2012 to 2019. As a comparison group, 50 healthy subjects (HC) were included. All subjects were caucasians. Diagnosis of IPF was made according to the ATS/ERS guidelines 2011 [11] and 2018 [12]. Two patients had familiar IPF. The comorbidity burden was assessed using the Charlson comorbidity Index (CCI), which scores 19 comorbidities and age to provide a total score between 0 and 37 [13].

### Definition of disease progression in ILD

Disease progression from baseline was defined as a deterioration of self-reported symptoms (worsening of dyspnoea, cough) and relative decrease in FVC > 10% pred., and/or a decrease in DLco ≥ 15% pred. and/or increase in existing or appearance of new densities compatible with IPF at HRCT [14, 15]. Otherwise, the patients were defined as stable. Acute exacerbation was defined according to the ATS/ERS consensus paper [16].

### DNA extraction and genotyping

Genomic DNA was extracted from peripheral blood leukocytes by using a silica-membrane- based nucleic acid purification kit (Qiagen DNA Mini Kit, Qiagen, USA), and stored at − 80 °C before use. Three SNPs at the chr11p15.5 locus were chosen for this analysis: rs35705950 (position chr11:1241221), rs5743890 (position chr11:1325829) and rs3750920 (position chr11:1309956).

Genotyping of the MUC5B rs35705950, TOLLIP rs3750920 and rs5743890 single-nucleotide polymorphism involved a commercially available SNP genotyping assay (TaqMan SNP Genotyping Assay) and the Applied Biosystems 7500 Fast RT-PCR System (Life Technologies Corp. Carlsbad, California, USA) as reported previously [4, 17, 18].

### Pulmonary function tests and blood gas analysis

Measurements including forced vital capacity (FVC), forced expiratory volume in one second (FEV_1_), total lung capacity (TLC), diffusing capacity of the lung for carbon monoxide (DLco), partial pressure of oxygen in arterial blood (PaO_2_), arterial carbon dioxide tension (PCO_2_), arterial oxygen saturation (SaO_2_) and alveolar-arterial oxygen gradient (AaDO_2_) were performed at the time of the blood sample collection. The protocol for pulmonary function measurements conformed to ATS recommendations [19]. Values were expressed as percentages of predicted normal values.

### Statistical analysis

Continuous variables were evaluated for a normal distribution with the Kolmogorov–Smirnov test. Parametric data are presented as mean ± SEM. Categorical variables are presented as either a percentage of the total or numerically, as appropriate. Spearman’s or Pearson’s correlation coefficient was obtained for correlations. Haplotype frequencies for multiple loci were estimated by means of HPlus v2.5 software for biallelic markers. The measures of pair-wise linkage disequilibrium between SNPs were calculated by using the expectation–maximization (EM) algorithm with SNPAlyze (Version 5.1; Dynacom Co., Ltd., Yokohama, Japan). Chi-square test and Fisher’s exact test were used to test for deviation from Hardy–Weinberg equilibrium (HWE).

Univariate and multivariate Cox proportional hazard regression model was conducted to study the independent effect of age, gender, smoking history, ethnicity, MUC5B and TOLLIP genotypes, and pulmonary function tests on the disease course. The Kaplan–Meier method with the log-rank test was used to analyze whether SNP genotypes were associated with the disease outcome. *p* values of < 0.05 were considered statistically significant. All statistical analyses were performed using SPSS 27.0 (SPSS Inc., Chicago, IL, USA).

## Results

### Characteristics of study subjects

Demographics and clinical characteristics of the studied subjects are shown in Table 1. Comorbidities are shown separately in Additional file 1: Table S1. Follow up time of IPF patients was 7.5 ± 4.5 years. Forty-five patients (72.5%) received antifibrotic treatment, 15 (24%) received N-acetylcysteine (NAC) monotherapy and 2 no treatment over time. Sixteen patients (26%) died and 12 (19%) underwent lung transplantation during follow-up. 19 (31%) patients experienced acute exacerbation. Median survival time, defined as time to death or lung transplantation, was 5.9 ± 3.2 years.

**Table 1:** Demographics and characteristics of the studied subjects

Variable	IPF (n = 62)	HC (n = 50)	*p*
Age (year)	63.5 ± 11	42 ± 2	< 0.001
Gender (M/F), n	43/8	37/13	0.228
Smoking habits (non/ex/current), n	9/40/4	33/7/10	< 0.001
Pack years, median (min–max)	28 (1–80)	15 (1–40)	0.018
BMI (kg/m^2^)	27 ± 4	25.5 ± 3	0.064
PaO_2_ (mmHg)	73 ± 12	–	–
FVC (% pred)	69 ± 21	–	–
DLco (% pred)	43 ± 14	–	–
*BAL cell differentials*		–	–
Total cells (× 10^4^ mL^−1^)	18 ± 3		
Macrophages (%)	69 ± 25		
Lymphocytes (%)	10 ± 10		
Neutrophils (%)	15 ± 23		
Eosinophils (%)	3.5 ± 3		
Antifibrotic treatment (yes/no)	45/17	–	–
Duration of antifibrotic treatment (months)	43 ± 16	–	–
Time to progression (months), median (IQR)	50 (34–68)	–	–
Time to death or lung transplant (months), median (IQR)	70 (40–95)	–	–
CCI* (score)	3.7 ± 1.7		

Otherwise indicated, values are expressed as mean ± SD

^*^Charlson comorbidity index

### MUC5B and TOLLIP SNPs allele and genotypes distribution

Allele and genotype distribution for each SNP in IPF and HC is shown In Table 2. MUC5B rs35705950, TOLLIP rs5743890 and rs3750920 alleles were all in HWE. The frequency of MUC5B rs35705950 minor allele (T) was significantly different between IPF patients and HC (*p* < 0.001). For TOLLIP rs5743890 and rs3750920 the frequency of minor alleles did not differ between IPF and HC (Table 2). Genotype distribution did not differ significantly between IPF and HC for the three SNPs studied.

**Table 2:** Genotype and allele distribution of the SNP rs35705950 of MUC5B, rs5743890 and rs3750920 of TOLLIP for IPF and HC subjects

Group	MUC5B rs35705950					*p*
	Genotype			Allele		
	G/G (%)	G/T (%)	T/T (%)	G (%)	T (%)	
HC	42 (84)	7 (14)	1 (2)	91 (91)	9 (9)	
HWE			0.304*			< 0.001**
IPF	23 (37)	35 (56)	4 (6)	81 (65)	43 (35)	
HWE			0.052*			

Group	TOLLIP rs5743890					*P*
	Genotype			Allele		
	T/T (%)	C/T (%)	C/C (%)	T (%)	C (%)	
HC	40 (80)	9 (18)	1 (2)	89 (89)	11 (11)	
HWE			0.568*			0.999**
IPF	48 (77)	14 (23)	0 (0)	110 (89)	14 (11)	
HWE			0.316*			

Group	TOLLIP rs3750920					*P*
	Genotype			Allele		
	C/C (%)	C/T (%)	T/T (%)	C (%)	T (%)	
HC	14 (28)	26 (52)	10 (20)	54 (54)	46 (46)	
HWE			0.741*			0.999**
IPF	15 (24)	37 (60)	10 (16)	67 (54)	57 (45)	
HWE			0.112*			

*p value of either chi-square test or Fisher’s exact test is indicated to determine the deviation from HWE

**Fisher’s exact test

Four major haplotypes with three polymorphic sites were established. The haplotype TTT occurred more frequently and GTT less frequently in IPF patients than HC (Additional file 1: Table S2).

Measures of pair-wise linkage disequilibrium between MUC5B and TOLLIP SNPs are shown in Additional file 1: Table S3. MUC5B rs35705950 and TOLLIP rs5743890 were in high linkage disequilibrium (D′-1, R^2^ 0.038, *p* = 0.0035).

### Correlations between SNPs and clinical characteristics

None of the SNP genotypes was associated with gender, BMI, smoking habits, lung function, BAL cell differentials or comorbidities at baseline (data not shown). No difference in the frequency of antifibrotic or NAC treatment was observed between genotypes.

### MUC5B und TOLLIP SNPs and pulmonary dysfunction at baseline and over time

Lung function tests at baseline in patients with TOLLIP rs5743890 C/T genotype (n = 14) did not differ from those in patients with T/T genotype (n = 48) (Additional file 1: Table S4). Patients with TOLLIP rs5743890 C/T genotype tended to have a greater decline in FVC, DLco % pred. and PaO2 mmHg per year compared to those with T/T genotype (Additional file 1: Table S4). No differences were seen in lung function tests at baseline or decline over time according to MUC5B and TOLLIP rs3750920 genotype (data not shown).

### Correlation between MUC5B and TOLLIP genotype and survival

**Fig. 1:**
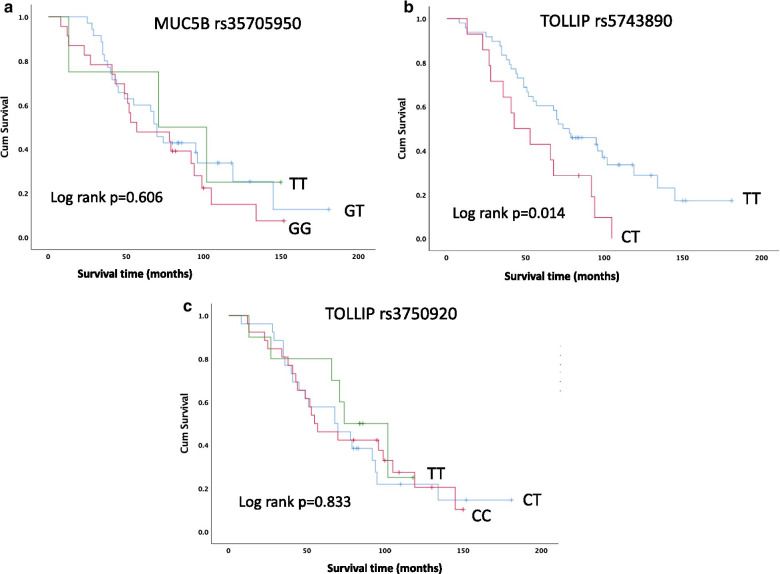
Kaplan–Meier analysis showing survival according to MUC5B rs35705950 (**a**), TOLLIP rs5743890 (**b**) and rs3750920 (**c**) genotype in IPF patients

The frequency of death or lung transplantation did not vary across MUC5B and TOLLIP genotypes. Kaplan–Meier analysis for MUC5B and TOLLIP rs3750920 did not show any correlation with survival, and median survival time did not vary according to genotype (Fig. 1a, c). Patients with TOLLIP rs5743890 C/T genotype had worse survival compared to T/T genotype, T being the major allele (*p* = 0.014) (Fig. 1b, Additional file 1: Table S4).

### Correlation between MUC5B and TOLLIP genotype and disease progression

Disease progression as defined in the methods occurred in a total of 37 (62%) of 60 patients where data were available. Median time to progression was 50 months (IQR: 34–68). No association was observed between MUC5B and TOLLIP rs3750920 genotype and disease progression.

**Fig. 2:**
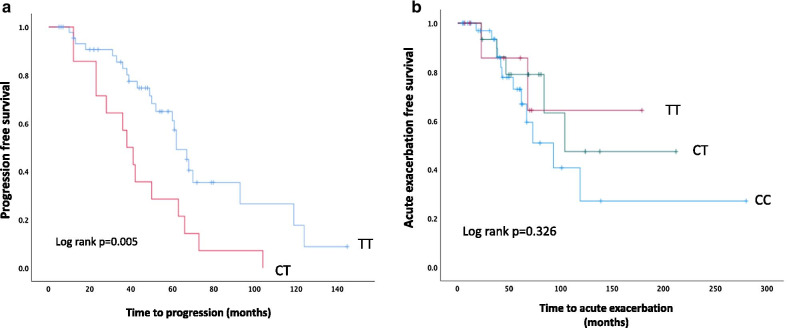
Kaplan–Meier analysis showing disease progression (**a**) and acute exacerbation (**b**) according to TOLLIP rs5743890 genotype in IPF patients

The frequency of disease progression significantly varied across TOLLIP rs5743890 genotype: all patients with C/T genotype had disease progression compared to 50% of patients carrying T/T (*p* = 0.001) (Additional file 1: Table S5) and median time to progression tended to be shorter in patients with the C/T genotype, 43.5 ± 23 months vs 63 ± 27 months compared to those with the T/T genotype (*p* = 0.058). Kaplan Kaplan–Meier analysis of disease progression according to TOLLIP rs5743890 genotype in IPF patients is shown in Fig. 2a.

### Correlation between MUC5B and TOLLIP SNP genotype and acute exacerbation

Acute exacerbation occurred in 19/62 (31%) patients. Median time to first acute exacerbation was 47 months (IQR: 38–73). 15 patients had AE after disease progression had started and 4 patients during a stable disease course. We did not analyze this small subgroup separately.

No association between MUC5B or TOLLIP genotype and frequency of acute exacerbation was seen (Additional file 1: Table S5). Whereas Kaplan–Meier analysis did not show any significant association between AE and TOLLIP rs5743890 genotype (Log rank *p* = 0.326), a tendency for an unfavorable effect of the minor allele can be observed (Fig. 2b).

### Uni and multivariate analysis for predictors of survival, disease progression and AE

We performed univariate and multivariate analysis by using Cox regression to investigate the role of several factors as predictors for survival and disease outcome. We did not find any association between MUC5B or TOLLIP rs3750920 and survival or AE (Table 3 and S6). Disease progression was found to be a strong predictor of AE in the univariate analysis (HR 4.627, CI 0.105–20.175, *p* = 0.041) but this association was not confirmed in the multivariate analysis (Additional file 1: Table S6). In the uni- and multivariate analysis for identifying predictors of death or lung transplantation, TOLLIP rs5743890 (C/T genotype) was the strongest one, followed by Dlco % pred., also after including a number of covariates in the model (HR 3.434, 95% CI 1.360–8.671, *p* = 0.009) (Table 3).

**Table 3:** Univariate and multivariate Cox proportional hazard model evaluating predictors of death or lung transplantation

Variables	β	HR	(95% CI)	*p* value
*Univariate analysis*				
Age at diagnosis (continuous)	0.030	1.030	0.976–1.088	0.282
Gender (male)	0.393	1.481	0.583–3.764	0.409
Smoking history (smoker)	0.543	1.721	0.672–4.404	0.258
CCI** (> 3)	− 0.178	0.837	0.280–2.498	0.749
BMI (continuous)	0.068	1.070	0.996–1.149	0.064
FVC % pred (continuous)	− 0.006	0.994	0.980–1.008	0.406
PaO_2_ mmHg (continuous)	0.103	1.108	0.989–1.241	0.076
DLco % pred (continuous)	0.006	0.966	0.942–0.990	0.006
Antifibrotic treatment (yes)	0.549	1.332	0.648–3.136	0.074
MUC5B (T allele)	− 0.615	0.541	0.069–4.250	0.559
TOLLIP rs5743890 (C/T genotype)	0.799	2.224	1.151–4.297	0.017
TOLLIP rs3750920 (T allele)	− 0.267	0.773	0.325–1.837	0.560
Multivariate analysis*				
TOLLIP rs5743890 (C/T genotype)	1.234	3.434	1.360–8.671	0.009
DLco (continuous)	− 0.071	0.931	0.898–0.966	0.001

*Model obtained by using backward conditional stepwise regression through 7 steps, including age, gender, smoking status, BMI, FVC, DLco and PaO2 as covariates

**Charlson Comorbidity Index

The results of uni- and multivariate analysis for predictors of disease progression are shown in Table 4. In the univariate analysis, FVC % pred. and TOLLIP rs5743890 C/T genotype were the strongest predictors of disease progression (Table 4) but in the multivariate analysis only TOLLIP rs5743890 C/T genotype was identified as a predictor of disease progression after including age, gender, BMI, smoking status and lung function at baseline as covariates in the multivariate model (HR 2.949, 95% CI 1.327–6.556, *p* = 0.008) (Table 4).

**Table 4:** Univariate and multivariate Cox proportional hazard model evaluating predictors of disease progression

Variables	β	HR	(95% CI)	*p* value
*Univariate analysis*				
Age at diagnosis (continuous)	0.025	1.026	0.991–1.062	0.152
Gender (male)	0.001	1.001	0.385–2.606	0.998
Smoking history (non smoker)	− 0.663	0.515	0.060–4.437	0.546
CCI** (> 3)	0.873	1.011	0.260–2.406	0.842
BMI (continuous)	0.028	1.029	0.951–1.113	0.481
FVC % pred (continuous)	0.018	1.018	1.002–1.034	0.027
PaO_2_ mmHg (continuous)	0.016	1.016	0.982–1.052	0.363
DLco % pred (continuous)	0.002	1.002	0.977–1.028	0.884
Antifibrotic treatment (yes)	0.669	1.602	0.952–3.004	0.068
MUC5B (T allele)	− 1.378	0.252	0.034–1.862	0.177
TOLLIP rs5743890 (C/T genotype)	0.923	3.517	1.276–5.964	0.008
TOLLIP rs3750920 (T allele)	− 0.136	0.873	0.290–2.626	0.809
Multivariate analysis*				
TOLLIP rs5743890 (C/T genotype)	1.082	2.949	1.327–6.556	0.008

*Model obtained by using backward conditional stepwise regression (11 steps), including age, gender, smoking status, BMI and antifibrotic treatment as covariates

**Charlson Comorbidity Index

## Discussion

In this study, we confirm that the MUC5B rs35705950 minor allele (T) is associated with IPF. The TOLLIP gene variant rs5743890 appears to correlate with survival and disease progression, therefore being of potential utility to stratify IPF patients at diagnosis.

The MUC5B promoter variant rs35705950 has been recognized as the dominant risk factor for developing (IPF) [7, 17, 20, 21]. A recent metanalysis encompassing 2733 IPF patients and 5044 controls from 8 different cohorts confirmed that the minor T allele is significantly and strongly associated with an increased risk of IPF compared with the G allele (odds ratio [OR] 4.85, 95% confidence interval [CI] 3.79–6.21) in an allele dose-dependent manner. The strength of this association was more remarkable in Caucasians [22]. In line with these previous studies, we found that the minor allele T (G/T and T/T genotype) is more frequent in IPF patients than in healthy controls (*p* < 0.0001). There was no difference with regard to age, gender or functional impairment at diagnosis between carriers or non-carriers of the minor allele T in our cohort. Borie et al. already reported similar results for French patients [23]. We did not observe any association of MUC5B variants with survival, disease progression or acute exacerbation. In previous studies the associations with survival were contradictory. A significant association of the minor allele T with improved survival has been described in sporadic IPF in Caucasians (significant reduction in 2-year cumulative incidence of death) [3] whereas an association with increased mortality was reported in Han patients [24]. A recent study from the Netherlands on a total of 170 IPF patients did not find an association of the minor allele T with survival in patients with sporadic IPF (N = 115), in contrast to MUC5B minor allele carriers with familial IPF (N = 55), who had a better survival (carriers 53 months vs non-carriers 37 months, *p* = 0.01) [25]. Consistently with previous studies [3, 26], Van der Vis et al. did not find any association of MUC5B minor allele with severity of lung function impairment at diagnosis both in sporadic and familiar IPF [27]. In 187 Han patients with IPF, carriers of MUC5B minor allele had significantly lower FVC and DLco values compared to those with homozygote major allele genotype (*p* < 0.0001 for both) [24]. Further, Stock et al. did not observe any association of MUC5B minor T allele with lung function impairment at diagnosis, but with a longer time to decline in FVC (HR 0.59, *p* = 0.052), while no association was seen with time to decline in DLco (*p* = 0.9) [26]. We did not find a significant association of the minor allele T with lung function impairment at diagnosis or decline over time. These findings are in line with a previous study from our group comparing the MUC5B polymorphism in German and Japanese IPF patients [18].

We did not find an association between TOLLIP SNPs alleles and IPF. This is in contrast to two previous genome-wide studies where the TOLLIP rs5743890 minor allele (C) was found to be protective and associated with reduced susceptibility to IPF [2, 20]. Intriguingly, in one of these studies, IPF patients carrying this protective minor allele had increased mortality in three different cohorts (on average HR 1.72 with 95% CI 1.24–2.38, *p* = 0.0012). In the lung tissue from these patients the expression of TOLLIP protein was reduced by 20% [2]. We also observed that patients carrying this minor allele had a reduced survival compared to those with the T/T genotype, with a remarkable difference of 20 months in median survival time. This association with survival was confirmed by multivariate analysis, after adjustment for a number of covariates. Consistently, patients with the C/T genotype had a higher rate of disease progression and tended to have a greater decline in FVC over time than patients with the T/T genotype. We could also demonstrate that the TOLLIP rs5743890 C/T genotype was the strongest predictor of disease progression in a multivariate analysis (HR 3.212, *p* = 0.003). So far, this is the first study showing an association of the minor allele (C) in TOLLIP rs5743890 with disease progression in IPF. Taken together, these data suggest a potential role of this SNP as a genetic biomarker for risk stratification of IPF patients in a real-world setting.

With respect to the other investigated TOLLIP SNP rs3750920, we did not find any significant association of the genotypes with survival or disease progression. However, a tendency for a protective role for AE of the minor allele (T) was observed (Fig. 2). The uni- and multivariate analysis did not confirm this association, probably due to the low number of events and to the fact that the majority of the AEs occurred as disease progression had already started, acting as a confounding factor. It may be speculated that mutations in the TOLLIP gene, encoding a protein whose expression levels contribute to varying degrees of human airway epithelial responses to infections [28], can increase the incidence of AEs which are mostly triggered by infections. This point needs further investigation,

Oldham et al. found that the TOLLIP rs3750920 polymorphisms may influence the response to NAC therapy in patients with IPF. Patients with the TOLLIP rs3750920 homozygote genotype for the minor allele may benefit from NAC therapy, whereas NAC may cause harm to patients carrying the homozygote genotype for the major allele [4]. Due to the low number of patients treated with NAC in our cohort (24%), we were not able to replicate these observations. We did not find any association of MUC5B or TOLLIP SNPs with clinical response to treatment with antifibrotic drugs.

The importance of TOLLIP in IPF is emphasized by the relationship between biological impact and potential disease outcome. Besides the well-known role in inflammation and autoimmunity by enhancing signaling pathways associated with IL-1β, TNF-a, IFN-b, IL-13, toll-like receptor (TLR) [29] and autophagy [30], a recent study revealed that TOLLIP protects bronchial epithelial cells from bleomycin-induced apoptosis [31]. A study investigating TOLLIP expression in lung tissue of IPF patients revealed that the TOLLIP expressing cells were macrophages, alveolar type II, and basal cells. A mechanistic assay using basal cells showed that TOLLIP was able to reduce mitochondrial ROS accumulation following bleomycin-induced mitochondrial damage of basal cells [31]. All these data together corroborate the hypothesis of a protective effect of TOLLIP in several cell populations against oxidative damage and fibrosis. This could be a possible explanation of the link between TOLLIP SNPs and worse outcome in IPF. Further validation in bigger cohorts is needed.

Despite the novel findings of this study, it has several limitations. First, the number of patients is rather low for a genetic study and a validation cohort was not included. We found for example, that alleles in MUC5B and TOLLIP rs5743890, despite the highest linkage disequilibrium, were differently distributed, suggesting a possible genetic instability of the locus or, simpler, that a larger number of subjects is needed to confirm allele distribution. Second, there was an imbalance in age and smoking habits between patients and healthy controls. Further, only two subjects had familial IPF, so that a comparison with sporadic IPF patients, as other studies did [27], was not possible in our cohort.

In conclusion, we confirm that the minor allele T at MUC5B rs35705950 is associated with IPF and that the minor allele C at TOLLIP rs5743890 is associated with an increased risk of death and disease progression. Although the results are promising, further validation in multi-center and multi-ethnic studies is needed.

## Supplementary Information

**Additional file 1**. Supplementary Material.

## Data Availability

The datasets used and/or analysed during the current study are available from the corresponding author on reasonable request.

## Abbreviations

AaDO_2_: Alveolar arterial oxygen gradient
BAL: Bronchoalveolar lavage
CCI: Charlson comorbidity index
COPD: Chronic obstructive pulmonary disease
DLco: Diffusing capacity of the lung for carbon monoxide
FVC: Forced vital capacity
GERD: Gastroesophageal reflux disease
GWAS: Genome wide association study
HRCT: High resolution computed tomography
ILD: Interstitial lung diseases
IPF: Idiopathic pulmonary fibrosis
KM: Kaplan–Meier analysis
MUC5B: Mucin 5B
OSAS: Obstructive sleep apnea syndrome
PaO_2_: Partial pressure of oxygen in arterial blood
ROC: Receiver operating characteristic
ROS: Reactive oxygen species
SNPs: Single nucleotide polymorphisms
TLC: Total lung capacity
TOLLIP: Toll interacting protein
