# Supplementary material for: Potential clinical utility of MUC5B und TOLLIP single nucleotide polymorphisms (SNPs) in the management of patients with IPF
Source: Orphanet J Rare Dis. 2021 Feb 27;16:111. doi: 10.1186/s13023-021-01750-3 (PMC7913255; doi:10.1186/s13023-021-01750-3)
Supplement: Supplementary file 1 — Additional file 1. Supplementary Material. [file 13023_2021_1750_MOESM1_ESM.docx]

**Supplemental Material**

**Table S1** Comorbidities observed at diagnosis in IPF subjects (frequency >10%)

|  | N=62 | % |
| --- | --- | --- |
| Coronary artery disease | 25 | 40 |
| Arterial hypertension | 22 | 35 |
| Diabetes | 15 | 24 |
| Hypercholesterinemia | 13 | 21 |
| OSAS | 12 | 19 |
| COPD | 10 | 16 |
| GERD | 8 | 13 |
| Pulmonary Hypertension | 7 | 11 |
| Other* | 6 | <10 |
| *lung cancer (2), liver cirrhosis (1), multiple sclerosis (1), psoriasis (2) | | |

**Table S2.** MUC5B, TOLLIP SNPs haplotype frequencies and association with IPF in the studied subjects.

| **Common**  **haplotypes** | **IPF**  **(freq.)** | **(S.E.)** | **HC**  **(freq.)** | **(S.E.)** | **Coeff.** | **(S.E.)** | **OR** | **CI** | **Z** | **p** |
| --- | --- | --- | --- | --- | --- | --- | --- | --- | --- | --- |
| **GTC** | 0.4487 | 0.044 | 0.5399 | 0.049 | - | - | 1.00 | - | - | - |
| **TTT** | 0.2939 | 0.038 | 0.0800 | 0.030 | -1.6141 | 0.575 | 0.20 | (0.06- 0.61) | -2.81 | 0.005 |
| **GTT** | 0.0915 | 0.023 | 0.2700 | 0.043 | 1.0693 | 0.463 | 2.91 | (1.18-7.21) | 2.31 | 0.021 |
| **GCT** | 0.0740 | 0.025 | 0.1000 | 0.032 | 0.0978 | 0.547 | 1.10 | (0.38-3.22) | 0.18 | 0.858 |
| *** | 0.0915 | - | 0.0100 | - | -2.2511 | 1.111 | 0.11 | (0.01,-0.93) | -2.03 | 0.043 |
|  |  |  |  |  |  |  |  |  |  |  |
| **Uncommon haplotypes*** |  |  |  |  |  |  |  |  |  |  |
| **TTC** | 0.0527 | - | 0.0000 | - |  |  |  |  |  |  |
| **GCC** | 0.0388 | - | 0.0000 | - |  |  |  |  |  |  |
| **TCT** | 0.0000 | - | 0.0099 | - |  |  |  |  |  |  |
| *One of each haplotype occurred no more than 3 times. | | | | | | | | | | |

**Table S3** Measures of pair-wise linkage disequilibrium between MUC5B rs35705950 and TOLLIP rs5743890, rs3750920 in the studied subjects.

| **Pair A** | **Pair B** | **D** | **D'** | **CI 95%** | **R^2^** | **X^2^** | **p** |
| --- | --- | --- | --- | --- | --- | --- | --- |
| rs35705950 | rs5743890 | -0.0259 | -1.0000 | -1.000 - -0.9980 | 0,038 | 8,5077 | 0.0035 |
| rs35705950 | rs3750920 | 0.0932 | 0.7435 | 0.5775 - 0.9096 | 0,1963 | 44,8449 | <0.0001 |
| rs5743890 | rs3750920 | 0.0435 | 0.7219 | 0.4685 - 0.9752 | 0,0769 | 16,3751 | <0.0001 |

**Table S4** Patients characteristics according to TOLLIP rs5743890 genotype in IPF subjects.

| **Variable** | **T/T (n=48)** | **C/T (n=14)** | **p** |
| --- | --- | --- | --- |
| **Gender** (m/f), n | 40/8 | 13/1 | 0.373* |
| **Age** (year) | 65±11 | 58±9 | 0.051 |
| **Smoking habits**  (non/ex/current), n | 9/33/3 | 0/12/0 | 0.133* |
| **BMI** (Kg/m^2^) | 26±4 | 28±1 | 0.553. |
| **FVC** (%pred) | 64±25 | 58±22 | 0.558 |
| **DLco** (%pred) | 42±14 | 44±12 | 0.733 |
| **PaO_2_** (mmHg) | 73±10 | 74±12 | 0.467 |
| **FVC decline** (% pred) / year | -6.8 ±3 | -11.1±5 | 0.066 |
| **DLCO decline** (% pred) / year | -6.2 ±2 | -12.3±10 | 0.137 |
| **PaO2 decline** (mmHg) / year | -0.8±4 | -2.9±3 | 0.048 |
| **Antifibrotic treatment** (yes/no) | 35/13 | 10/4 | 0.912* |
| **Duration of antifibrotic treatment** (months) N=45 | 43±16 | 42±16 | 0.895 |
| **Time to progression,** (months) N=37 | 62.5±35 | 44±25 | 0.056 |
| **Time to death or lung transplant** (months) N= | 76±39 | 55±29 | 0.042 |
| **CCI (**score) | 4±2 | 3±2 | 0.948 |
| **CCI >3**, n (yes/no) | 29/19 | 4/10 | 0.036* |
| Otherwise indicated, values are expressed as mean±SD.  Charlson comorbidity index  *calculated by using Chi-Square test | | | |

**Table S5.** Frequency of disease progression and first acute exacerbation (AE) according to SNPs genotype in IPF patients.

|  |  | **Progression*** | | **AE-IPF** | |
| --- | --- | --- | --- | --- | --- |
| SNP | Genotype | Yes (N=37) | No (N=23) | Yes (N=19) | No (N=43) |
| **MUC 5B rs35705950** | G/G | 13 | 8 | 4 | 13 |
|  | G/T | 23 | 12 | 10 | 20 |
|  | T/T | 1 | 3 | 1 | 3 |
| **TOLLIP rs5743890** | C/T | 14** | 0 | 6 | 8 |
|  | T/T | 23 | 23 | 13 | 35 |
| **TOLLIP rs3750920** | C/C | 7 | 7 | 5 | 10 |
|  | C/T | 24 | 12 | 12 | 25 |
|  | T/T | 6 | 4 | 2 | 8 |
| * defined as decline of ≥ 10% in FVC over two follow up visits (data available in 60 pts.)  ** Pearson Chi-Square = 11.351, p= 0.001 vs other genotypes | | | | | |

**Table S6.** Univariate Cox proportional hazard model evaluating predictors of acute exacerbation.

| **Variables** |  | **HR** | **(95% CI)** | **p value** |
| --- | --- | --- | --- | --- |
| **Univariate analysis*** |  |  |  |  |
| **Age** **at diagnosis** (continuous) | 0.039 | 1.040 | 0.994-1.089 | 0.091 |
| **Gender** (male) | -0.937 | 0.392 | 0.051-3.002 | 0.367 |
| **Smoking history** (nonsmoker) | 0.010 | 1.010 | 0.363-2.809 | 0.985 |
| **CCI**** (>3) | 0.004 | 0.831 | 0.194-1.705 | 0.087 |
| **BMI** (continuous) | -0.005 | 0.995 | 0.879-1.126 | 0.932 |
| **FVC** % pred (continuous) | 0.002 | 1.002 | 0.981-1.024 | 0.840 |
| **PaO_2_** mmHg (continuous) | 0.005 | 1.005 | 0.967-1.044 | 0.808 |
| **DLco** % pred (continuous) | -0.020 | 0.981 | 0.943-1.020 | 0.329 |
| **Disease progression***** | 1.532 | 4.627 | 0.105-20.175 | 0.041 |
| **Antifibrotic treatment** (yes) | 0.669 | 1.602 | 0.952-3.004 | 0.068 |
| **MUC5B** (T allele) | -0.252 | 0.777 | 0.101-5.964 | 0.777 |
| **TOLLIP rs5743890 (**C/T genotype**)** | 0.307 | 1.360 | 0.511-3.619 | 0.538 |
| **TOLLIP rs3750920 (**T allele**)** | -0.316 | 0.729 | 0.168-3.168 | 0.673 |
| **Multivariate analysis** |  |  |  |  |
| **Disease progression** | 1.047 | 2.850 | 0.237-34.327 | 0.523 |
| *Charlson Comorbidity Index  **Defined as a deterioration of self-reported symptoms (worsening of dyspnea, cough) and relative decrease in FVC >10% pred., and/or a decrease in DLco ≥15% pred. and/or increase in existing or appearance of new densities compatible with IPF at HRCT. | | | | |
